# Optimized enantioselective (S)-2-hydroxypropiophenone synthesis by free- and encapsulated-resting cells of *Pseudomonas putida*

**DOI:** 10.1186/s12934-023-02073-7

**Published:** 2023-05-03

**Authors:** Reihaneh Kordesedehi, Mohammad Ali Asadollahi, Azar Shahpiri, Davoud Biria, Pablo Iván Nikel

**Affiliations:** 1 Department of Biotechnology, Faculty of Biological Science and Technology, University of Isfahan, Isfahan, Iran; 2 Department of Biotechnology, College of Agriculture, Isfahan University of Technology, Isfahan, Iran; 3 The Novo Nordisk Foundation Center for Biosustainability, Technical University of Denmark, Kongens Lyngby, Denmark

**Keywords:** αHydroxyketones, 2-Hydroxypropiophenone, *Pseudomonas putida*, Benzoylformate decarboxylase (BFD), Cell immobilization, Biotransformation

## Abstract

**Background:**

Aromatic α-hydroxy ketones, such as *S*-2-hydroxypropiophenone (2-HPP), are highly valuable chiral building blocks useful for the synthesis of various pharmaceuticals and natural products. In the present study, enantioselective synthesis of 2-HPP was investigated by free and immobilized whole cells of *Pseudomonas putida* ATCC 12633 starting from readily-available aldehyde substrates. Whole resting cells of *P. putida*, previously grown in a culture medium containing ammonium mandelate, are a source of native benzoylformate decarboxylase (BFD) activity. BFD produced by induced *P. putida* resting cells is a highly active biocatalyst without any further treatment in comparison with partially purified enzyme preparations. These cells can convert benzaldehyde and acetaldehyde into the acyloin compound 2-HPP by BFD-catalyzed enantioselective cross-coupling reaction.

**Results:**

The reaction was carried out in the presence of exogenous benzaldehyde (20 mM) and acetaldehyde (600 mM) as substrates in 6 mL of 200 mM phosphate buffer (pH 7) for 3 h. The optimal biomass concentration was assessed to be 0.006 g dry cell weight (DCW) mL^− 1^. 2-HPP titer, yield and productivity using the free cells were 1.2 g L^− 1^, 0.56 g 2-HPP/g benzaldehyde (0.4 mol 2-HPP/mol benzaldehyde), 0.067 g 2-HPP g^− 1^ DCW h^− 1^, respectively, under optimized biotransformation conditions (30 °C, 200 rpm). Calcium alginate (CA)–polyvinyl alcohol (PVA)-boric acid (BA)-beads were used for cell entrapment. Encapsulated whole-cells were successfully employed in four consecutive cycles for 2-HPP production under aerobic conditions without any noticeable beads degradation. Moreover, there was no production of benzyl alcohol as an unwanted by-product.

**Conclusions:**

Bioconversion by whole *P. putida* resting cells is an efficient strategy for the production of 2-HPP and other α-hydroxyketones.

**Graphical abstract:**

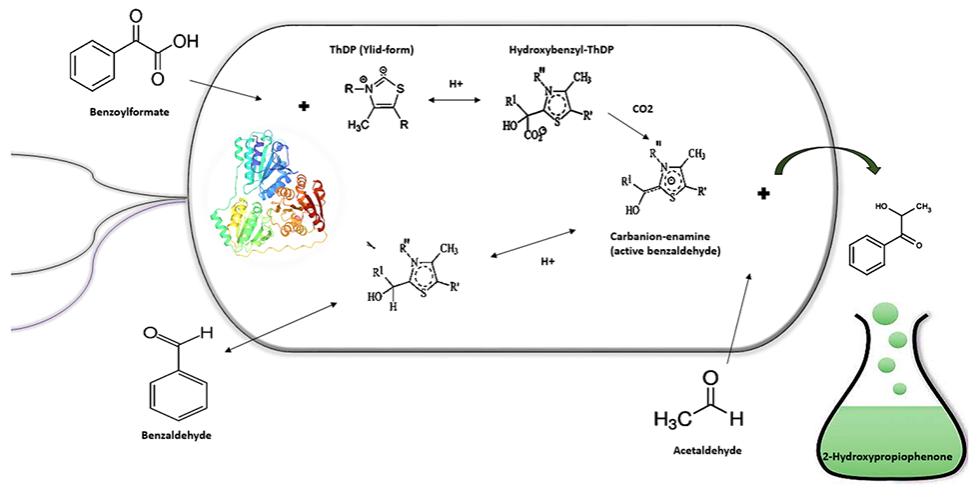

## Background

Optically active α-hydroxy ketones (also called acyloins) are commercially attractive compounds with unique features which allow meeting their great applications in the pharmaceutical and chemical industry [1, 2]. Phenylacetylcarbinol (S/R-PAC) [3, 4] and *S*-2-hydroxypropiophenone (2-hydroxy-1-phenyl-1-propane, 2-HPP) are among the important α-hydroxyketones. 2-HPP is an essential intermediate in the stereoselective synthesis of diols. For instance, 1-phenylpropane-1,2-diol (PPD), an important agent and versatile building block in pharmaceutical industry, can be obtained by subsequent oxidoreduction of 2-HPP using alcohol dehydrogenase [5, 6]. Different α-hydroxy ketone derivatives can be formed by numerous chemical synthetic routes [7]. However, these traditional approaches suffer from harsh reaction conditions with multiple reaction steps, low yield, production of unwanted by-products and poor enantioselectivity [2, 8, 9]. It is possible to overcome these hurdles with the assistance of biocatalysts, in the form of either pure enzymes or whole cells, for industrial synthesis [10–14]. In recent years, many studies have focused on cross acyloin condensation reactions of two inexpensive aromatic/aliphatic aldehydes [15] or the corresponding α-keto acids catalyzed by carboligation activity of thiamine pyrophosphate (ThDp)-linked enzymes, yielding α-hydroxy ketones in high yields and high enantioselectivity [16–20]. The well-established class of this family are versatile ThDp and Mg^2+^ dependent lyases that include pyruvate decarboxylase (PDC), benzaldehyde lyase (BAL) and benzoylformate decarboxylase (BFD) [21] from *Saccharomyces cerevisiae, Pseudomonas fluorescens* and *Pseudomonas putida*, respectively [22–24]. Most of these enzymes are (R)-selective, and the only known exception is BFD, which produces aromatic (*S*)-configured α-hydroxy ketones [15, 25, 26]. Valinger et al. [27], for instance, examined the continuous synthesis of 2-HPP from benzaldehyde and acetaldehyde by BFD in an enzyme ultrafiltration membrane reactor (UFMR) and three different laminar flow microreactors (MRs). In contrast to UFMR, all tested MRs supported complete conversion of benzaldehyde at a processing time longer than 4 min. Also, higher biocatalytic productivity numbers were obtained for the MRS comparing to the UFMR.

Most of the reports available in the literature have been focused on 2-HPP production by crude *P. putida* cell extracts or purified BFD enzyme preparations [15, 28, 29]. Nevertheless, several drawbacks including the need to costly cofactors and tedious and time-consuming enzyme purification processes have limited large-scale application of enzymatically catalyzed synthesis of 2-HPP [15]. As compared to pure enzymes, utilizing whole-cell biocatalysts in biotransformation processes offers several advantages, including higher catalyst stability and efficiency, co-enzyme regeneration, multi-enzyme reactions in one strain and the possibility of using inexpensive substrates [30, 31]. Using metabolically active, resting cells as biocatalysts provides an alternative mode of production in which microbial cell proliferation and product formation phases are separated from carbon source consumption for biomass [32, 33]. Pyo et al. [34] recently reported on the synthesis of adipic acid by oxidation of 1,6‑hexanediol with high yield using resting cells of *Gluconobacter oxydans*. Domínguez de María et al. [35] reported that the direct use of recombinant *Escherichia coli* resting cells containing over-expressed BFD and BAL with a biphasic reaction medium could be a suitable strategy for high-yield production of (*S*)/(*R*)-2-HPP. *P. putida* is a ubiquitous and interesting microbial platform for industrial biotechnology applications [36, 37]. *P. putida* strain ATCC 12633 can grow on racemic (D/L)-mandelic acid as the sole carbon and energy substrate [38]. Mandelic acid can be converted to benzoic acid through the action of five enzymes and finally assimilated through the β-ketoadipate pathway and tricarboxylic acid (TCA) cycle. Benzoylformate is decarboxylated to benzaldehyde and CO_2_ by BFD, the third enzyme in the mandelate pathway [39]. Carboligase activity, a side reaction catalyzed by BFD, is responsible for the synthesis of 2-HPP [15, 25, 40].

PpBFD can produce different 2-HPP analogs by acting on diverse α,β-unsaturated aromatic aldehydes and acetaldehyde as donor and acceptor substrates, respectively [41, 42]. Few studies reported 2-HPP production by whole cells using benzaldehyde instead of benzoylformate as a donor molecule. Among them, the production of 2-HPP by whole cells of *P. putida* by Wilcocks et al. [18] used benzoylformate and acetaldehyde. However, since the biotransformation conditions were not optimized, benzyl alcohol and benzaldehyde also accumulated as by-products associated with 2-HPP production. Here, we focused our interest on the fact that whole *P. putida* cells grown in the presence of mandelic acid are able to biotransform aldehydes to 2-HPP with no added enzymes or cofactors. Furthermore, since *P. putida* cells can adapt to chemical stresses and harsh environmental conditions [43–46], aldehyde substrates can be tolerated better in comparison with other microbial hosts. Hence, this approach leads to a low-cost and efficient biocatalyst preparation and meets the requirements for industrial production of α-hydroxy ketones.

On the other hand, microencapsulation of the whole cells in a non-toxic, porous and spherical matrix is a promising technique for the immobilization and reuse of cells [47]. Calcium alginate (CA) is a suitable matrix that maintains the survival and activity of microbial cells [48, 49]. The surface and mechanical properties of CA are improved by its combination with polyvinyl alcohol (PVA), boric acid (BA) and CaCO_3_ [50]. In several studies, immobilized resting cells were used for microbial bioconversions [51]. In one study, rhamnolipid production by *P. nitroreducens* immobilized on CA beads and under resting cell conditions was investigated. The highest rhamnolipid titer of 5.6 g L^− 1^ was obtained using sucrose as carbon source at pH 8 and 35 °C [52]. Also, *P. aeruginosa* immobilized resting cells in CA beads can successfully be used for the bioconversion of tyrosol into hydroxytyrosol for at least four batches [53]. In the present work, resting cells of *P. putida* ATCC 12633 were employed to efficiently enhance 2-HPP production and prevent the initial metabolization of benzaldehyde without its participation in the biotransformation reaction. Then, reaction conditions were optimized to obtain higher yields. Moreover, enhanced 2-HPP production of *P. putida* was examined by immobilizing resting cells in CA-PVA-BA beads.

## Results and discussion

### Optimizing the BFD-catalyzed synthesis of 2-HPP in a resting cells-based bioprocess

Biotransformation is an efficient tool for the synthesis of different chiral α-hydroxy ketones. Bioconversion can be performed via growing cells, resting cells, partially purified or thoroughly purified enzymes. The industrial production of PAC is currently done using growing yeast cells in a process where the exogenous benzaldehyde is condensed with acetaldehyde using the carboligation activity of PDC enzyme [54]. A similar process was also examined applying growing *P. putida* cells for a fermentative 2-HPP synthesis in the presence of aldehyde substrates in this study. However, this strain did not have the ability to produce 2-HPP coupled to growth as expected. Since high concentrations of growth-inhibitory substrates could cause some toxicity problems in actively growing cells [55, 56]. The mandelate pathway also needs to be induced in the presence of mandelic acid and employing growing biocatalysts would not be particularly useful. Moreover, the microbial transformation of benzaldehyde and acetaldehyde to 2-HPP by *P. putida* growing cells may lead to the conversion of benzaldehyde to benzoate by benzaldehyde dehydrogenase in the mandelate pathway even prior to BFD induction [57]. So, it was envisioned that the bioconversion with *P.putida* in a resting cells process could be a better choice. Acyloin synthesis by resting cells is a mode of production in which there is no cell multiplication. Nevertheless, these cells are able to carry out the biotransformation using the enzymes and active cell metabolism [58]. To this end, biotransformation reactions were conducted utilizing *P. putida* whole resting cell biocatalysts as enzyme sources. Cells in *Pseudomonas* mineral medium (PMM) were supplemented with 3 g L^− 1^ ammonium mandelate and 1 g L^− 1^ yeast extract to completely induce BFD enzyme in cells. Cells were harvested in the middle of the exponential growth phase (OD_600_ 0.9-1) by centrifugation, washed and resuspended in phosphate buffer, and then incubated aerobically at 30 °C. Under these conditions, biomass duplication was prevented. So, the benzoin condensation reaction of *P. putida* resting cells was conducted in a batch synthesis in the presence of aldehydes without further processing. The reduction in the substrates concentration and the accumulation of 2-HPP in the reaction medium were monitored by frequent sample analysis by GC. Several parameters involved in reaction performance including substrate concentration, biomass concentration, reaction time, and pH were optimized to achieve the highest possible productivity and enantiomeric purity [15, 29, 40, 59].

### Optimization of acetaldehyde concentration

**Fig. 1:**
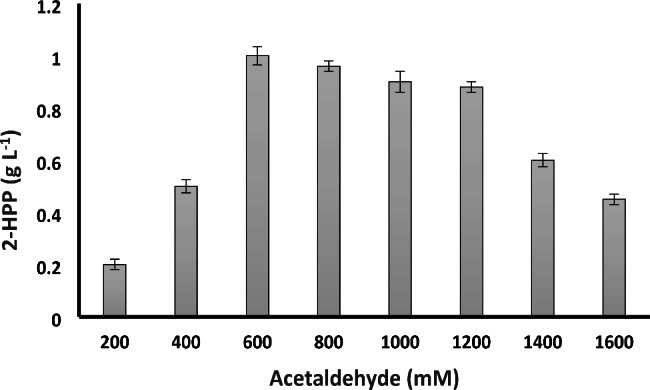
Effect of acetaldehyde concentration on the biotransformation efficiency. The experiments were carried out in a 100 mL Erlenmeyer flask containing fixed benzaldehyde concentration (50 mM) and varying concentrations of acetaldehyde at 30 °C and 200 rpm. Bioconversion was carried out aerobically at 30 °C and pH 7 with shaking at 200 rpm. Data are results of triplicate experiments ± SD

The cross condensation of benzaldehyde and acetaldehyde leads to the formation of S-2-HPP through carboligase activity of the BFD enzyme from *P. putida*. Acetaldehyde concentration and ratio of acetaldehyde to benzaldehyde concentration are key parameters affecting the biotransformation reaction and are prerequisites for the successful setup of a stable process catalyzed by BFD. Therefore, at a fixed benzaldehyde concentration of 50 mM, varied concentrations of acetaldehyde from 200 mM up to 1600 mM were examined. As shown in Fig. 1, increasing acetaldehyde concentration from 200 to 600 mM was concomitant with two-fold increase in the 2-HPP titer. High concentrations of acetaldehyde up to 600 mM can decrease the production of benzyl alcohol since acetaldehyde can deactivate oxidoreductase enzymes responsible for the conversion of benzaldehyde to benzyl alcohol [59]. However, further increase in acetaldehyde concentration negatively affected 2-HPP production probably due to irreversible inhibition of BFD by acetaldehyde [29, 60]. Besides, at acetaldehyde concentrations exceeding 600 mM, substrates were not used completely and more than 65% of benzaldehyde remained unconsumed. Park et al. [61] studied production of benzaldehyde by free and immobilized whole-cell benzoyl formate decarboxylase. They also investigated the effect of acetaldehyde added to the reactant mixture containing 100 mM (15 g L^− 1^) benzoylformate on the bio-products produced by a whole-cell enzyme. It was shown that a concentration of 2.73 g L^− 1^ 2-HPP was produced in the presence of 800 mM acetaldehyde as a co-reactant. The amount of benzaldehyde and benzyl alcohol produced were 5.13 and 0.3 g L^− 1^, respectively, and TPP, the expensive coenzyme of BFD, had to be added to the reactant mixture.

### Optimization of biotransformation duration

**Fig. 2:**
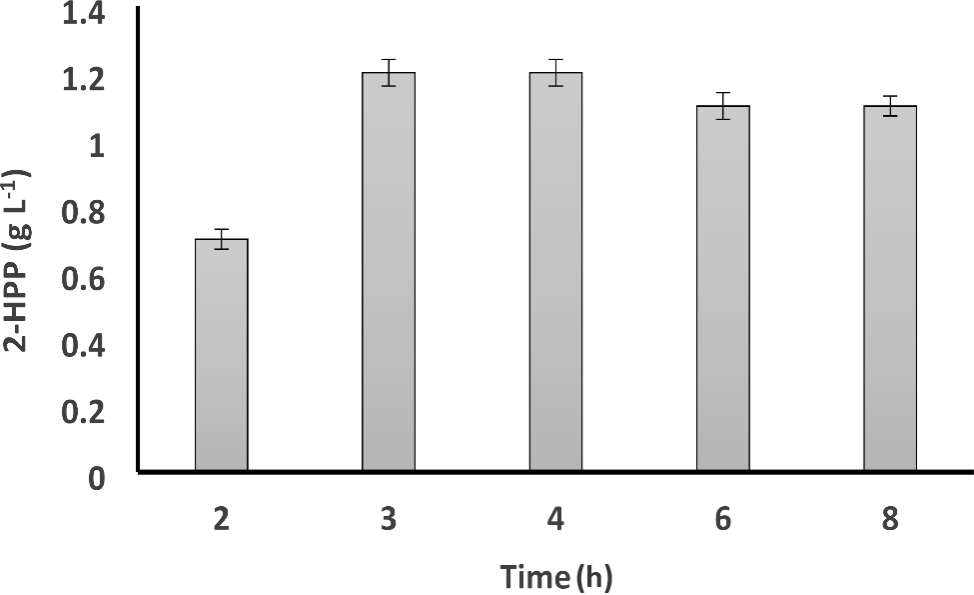
Effect of reaction time on 2-HPP formation. Reactions were performed in a 100 mL Erlenmeyer flask containing 50 mM benzaldehyde and 600 mM acetaldehyde at 30 °C and 200 rpm. Data are results of triplicate experiments ± SD

The effect of biotransformation time on the production of 2-HPP was studied. 2-HPP titers after 2 and 3 h were 0.7 g L^− 1^ and 1.2 g L^− 1^, respectively. However, lower 2-HPP were obtained over more prolonged time periods (Fig. 2). Therefore, biotransformation duration of 3 h was considered as the optimum time for further experiments.

### Optimization of biomass concentration

**Fig. 3:**
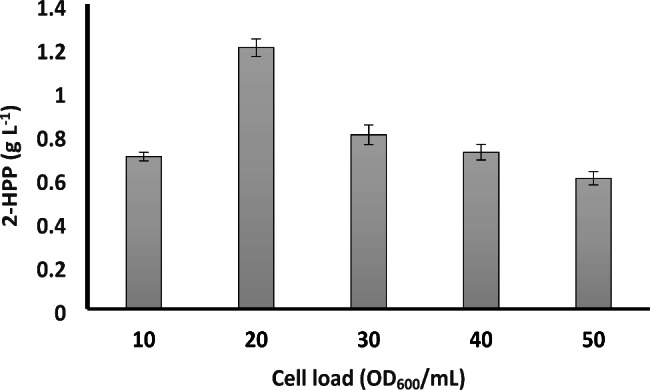
2-HPP production after 3 h with different cell concentrations. Reactions were performed in a 100 mL Erlenmeyer flask containing 50 m M benzaldehyde and 600 mM acetaldehyde at 30 °C and 200 rpm. Data are results of triplicate experiments ± SD

The initial cell concentration is one of the most critical parameters in the biotransformation of benzaldehyde and acetaldehyde to 2-HPP. Hence, the biotransformation reaction was set up at varying biomass concentrations to find the optimum value. Figure 3 represents the impact of whole-cell concentration on 2-HPP formation.

This figure shows that maximum 2-HPP titers were achieved at OD_600_ 20 that is equivalent to cell concentration of 0.033 g wet cell weight (WCW)/mL [0.006 g dry cell weight (DCW)/mL]. The yield of 2-HPP is not correlated to the increase in biomass concentration and 2-HPP production was not improved in a linear pattern proportional to the increase in cell load. This is probably due to the degradation of 2-HPP by the cell metabolism. We also speculated that at high biomass concentrations, cells demand a higher oxygen level. Similar results were obtained with resting cells of *Salinivibrio costicula* GL6 [62], *Halomonas elongata* [63], and *P. fluorescences* strain BF13 [64]. Furthermore, unwanted benzaldehyde side reaction leading to the formation of benzyl alcohol as a by-product was promoted with cell load surplus. Therefore, it was decided to use a cell load of 0.033 g of wet cell weight (WCW)/mL (corresponding to 0.006 g DCW/mL) in the following experiments to keep the by-product concentration at the lowest levels. Wilcocks et al. [18] demonstrated 2-HPP production from benzoylformate using acetaldehyde as the co-substrate with whole induced cells of *P. putida* for the first time. Although whole cells yielded less 2-HPP than crude cell extracts, a maximum 2-HPP concentration of 4.5 g L^− 1^ was achieved by 0.015 g DCW/mL of whole cells in the presence of 15 g L^− 1^ benzoylformate as the main substrate after 2 h of reaction. Furthermore, benzaldehyde (4.2 g L^− 1^) and benzyl alcohol (0.29 g L^− 1^) were formed as by-products in this biotransformation condition. Thiamine PP_i_ and magnesium chloride were also used for all the reaction conditions tested, including cell extracts and whole cells. Since the price of the α-ketoacid is usually higher than the price of aldehydes, this biotransformation reaction is relatively economical and cost-effective due to the use of inexpensive benzaldehyde instead of expensive and infrequent benzoylformate as the main substrate.

### Optimization of benzaldehyde concentration

**Fig. 4:**
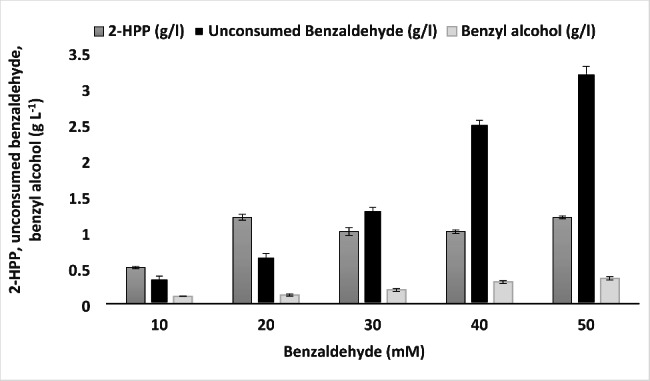
Effect of benzaldehyde concentration on 2-HPP formation, unconsumed benzaldehyde and benzyl alcohol production. Cell concentration, acetaldehyde concentration, and reaction time were set to 0.033 g WCW/mL, 600 mM and 3 h, respectively. Data are results of triplicate experiments ± SD

We wanted to test if increasing benzaldehyde concentration may yield higher product titers. However, high benzaldehyde concentrations could be toxic to the cells [65]. Therefore, finding optimum benzaldehyde concentration is vital. The results in Fig. 4 indicate that complete depletion of benzaldehyde did not occur in any of the reactions. Nevertheless, further experiments revealed that benzaldehyde was consumed entirely by increasing the reaction time up to 20 h but it may not be economically justified to continue the process for such prolonged times. As shown in Fig. 4, maximum 2-HPP titer was observed at 20 mM benzaldehyde. Interestingly, the minimum accumulation of benzyl alcohol as the main by-product was measured at benzaldehyde concentrations of 10 and 20 mM (Fig. 4).

It has been demonstrated that the ratio of benzaldehyde/acetaldehyde is important to form 2-HPP in an effective cross acyloin reaction. Higher concentrations of acetaldehyde in presence of lower benzaldehyde concentration is needed to produce 2-HPP with high yield [25]. Demir et al. [40] reported that 35-fold excess of acetaldehyde to benzaldehyde resulted in the formation of more 2-HPP since it prevents benzoin formation from homocoupling of two benzaldehyde molecules catalyzed by BFD. Our results showed maximum 2-HPP production at benzaldehyde and acetaldehyde concentrations of 20 mM and 600 mM, respectively. Applying an excess of benzaldehyde (30–40 and 50 mM) in relation to optimal acetaldehyde (600 mM) would mean that full conversion to 2-HPP cannot be achieved. Furthermore, unwanted self-condensation of benzaldehyde leading to the formation of R-benzoin and benzyl alcohol formation is promoted with a benzaldehyde surplus. Therefore, the optimum acetaldehyde to benzaldehyde concentration ratio was considered as 30.

### Optimization of pH

**Fig. 5:**
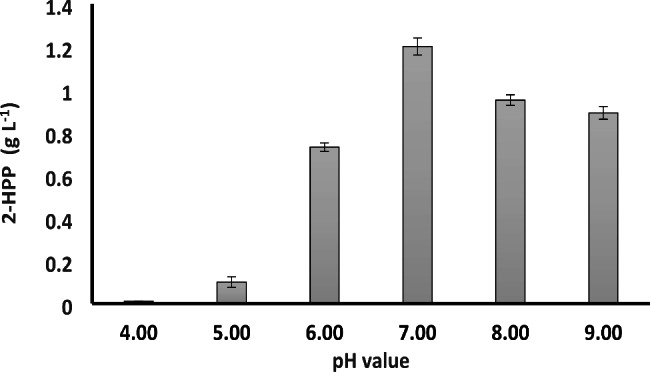
2-HPP profile at varying pH values. The reaction was performed at 20 mM benzaldehyde, 600 mM acetaldehyde, 0.033 g WCW/mL, 30 °C, and 200 rpm for 3 h. Data are results of triplicate experiments ± SD

Figure 5 demonstrates the effect of pH on 2-HPP titer. Highest 2-HPP concentrations were obtained at pH 7. Lower pH values resulted in a sharp decrease in 2-HPP production and reached almost zero at pH values below 5. On the other hand, pH values higher than 7 also had a negative impact on 2-HPP production.

### 2-HPP production using immobilized ***P. putida*** cells

The synthesis of 2-HPP starting from benzaldehyde and acetaldehyde has been examined by covalently immobilized BFD on magnetic epoxy attached magnetic nanoparticles. The activity of the immobilized BFD was determined to be 53% related to the activity of the free enzyme. The immobilized biocatalyst retained 95% of its original activity after five reaction cycles [66].

It is proven in several investigations that both aldehydes, benzaldehyde and acetaldehyde, have an inactivating effect on the activity of PpBFD. However, the inactivating impact of benzaldehyde is much higher in comparison to acetaldehyde [67, 68]. On the other hand, it would be better to use higher acetaldehyde concentration to achieve higher reaction rates. So, the simplest approach to overcome these limitations would be the use of immobilized whole cells.

**Fig. 6:**
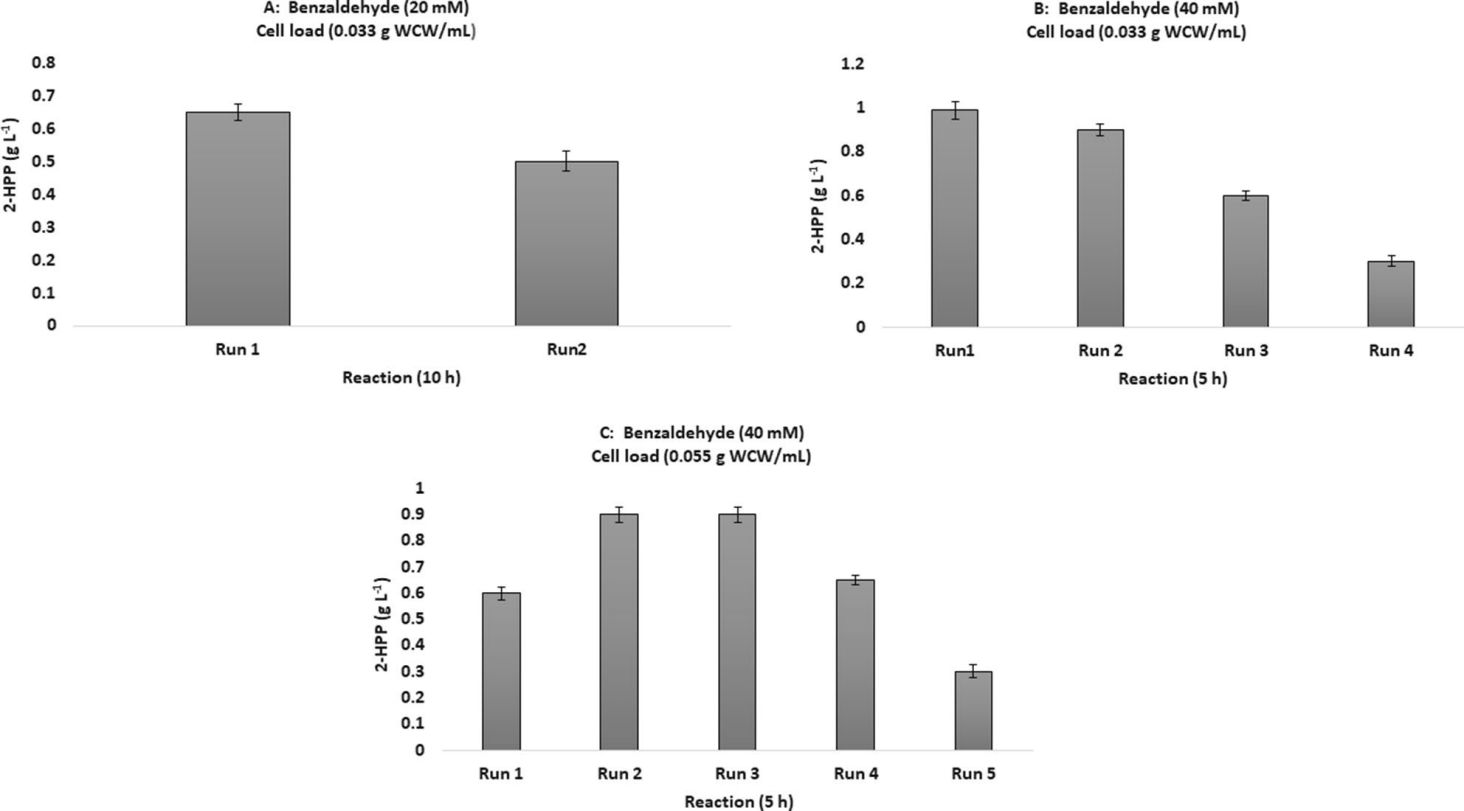
The profile of 2-HPP production by the encapsulated *P. putida*. A: 2-HPP production by a benzaldehyde concentration of 20 mM and with 0.033 g WCW/mL of immobilized cells load. B: 2-HPP production by a benzaldehyde concentration of 40 mM and with 0.033 g WCW/mL of immobilized cells load. C: 2-HPP production by a benzaldehyde concentration of 20 mM and with 0.055 g WCW/mL of immobilized cells load. Biotransformation was carried out aerobically with a constant acetaldehyde concentration of 600mM, 200 beads in 6 mL buffer at 30 ^°^C and pH 7 with shaking at 200 rpm. Data are results of triplicate experiments ± SD

Immobilization of cells enables recovery and reuse of *P. putida* cells for several cycles; thereby reducing the costs associated with 2-HPP production. Another merit to this immobilization system is that 2-HPP is produced purely without any terminal by-product formation since 2-HPP accumulated in the beads can inhibit alcohol dehydrogenases responsible for the conversion of benzaldehyde to benzyl alcohol [61, 69].Cells immobilized in CA-PVA produced 0.6 g L^− 1^ 2-HPP in the first cycle and 0.44 g L^− 1^ in the second cycle using 20 mM benzaldehyde and 600 mM acetaldehyde with 200 beads after 10 h (Fig. 6-a). Increasing the benzaldehyde concentration to 40 mM resulted in the production of 0.9 g L^− 1^ 2-HPP in the first and second cycles after 5 h (Fig. 6-b). However, 2-HPP production decreased to 0.6 g L^− 1^ and 0.3 g L^− 1^ in the third and fourth cycles, respectively (Fig. 6-b). Increasing the biomass load from 0.033 g WCW/mL to 0.05 g WCW/mL improved total 2-HPP production from 2.8 g L^− 1^ to 3.3 g L^− 1^ during successive cycles (Fig. 6-c). Although the residual activity of immobilized catalyst decreased gradually with the increasing number of reaction cycles, with the immobilized cells several biotransformation cycles can be repeated quickly without having to grow cells each time.

## Conclusion

Although enlargement of the ThDP-dependent enzymes in the toolbox [70] has allowed production of a number of α-hydroxy ketones, there are certain products which are still challenging to produce. One of the best examples is the production of the (*S*)-enantiomers which are not easily accessible by chemical synthesis approaches [5, 71]. 2-HPP is a highly valuable building block, and also there is a growing interest in the potential use of this α-hydroxy ketone as a feedstock for the biocatalysis into other value-added pharmaceutical products and intermediates [72]. Biotransformation process for the synthesis of 2-HPP has already been attempted in several studies mainly using cell crude extracts or purified enzymes. Wachtmeister et al. [5] applied a carboligation reaction mediated by recombinant lyophilized *E. coli* cells containing BFD variant L461A catalyst to provide 2-HPP with yield of 82% in the presence of 500 mM of benzaldehyde and 89 mM acetaldehyde in microaqueous MTBE and 1 M TEA buffer. Although there have been reports on the production of aromatic and heterocyclic aldehydes by *P. putida* ATCC 12633 under resting cell conditions [73, 74], there is no report on 2-HPP production by *P. putida* native resting biocatalysts as an alternative for biotransformation reactions. Inspired by those investigations, we used *P. putida* resting cells with the highest carboligase activity for the coupling of benzaldehyde and acetaldehyde to produce 2-HPP by whole-cell catalysis. On the one hand the usage of whole resting cells circumvents the time-consuming enzyme purification as well as addition of expensive cofactors (here ThDP and MgCl2). Additionally, catalyst and product workup is facilitated by the application of immobilized cells. On the other hand, the employment of whole cells very effectively overcomes stability problems of biocatalysts in unconventional media enabling the addition of extremely high substrate loads (up to 500 mM in our example for acetaldehyde) and is therefore an easy and effective approach to fulfill the requirements for translation to industrial application.

PpBFD can use two different types of donor substrates, aldehydes and the α-ketoacids for the carboligation. Nonetheless, no whole native cell biocatalyst is reported to selectively access 2-HPP starting from benzaldehyde and acetaldehyde as main substrates. To the best of our knowledge, this is the first report of the application of whole resting cells of *P. putida* for BFD-catalyzed formation of 2-HPP proceeded with a yield of 0.56 g 2-HPP/g benzaldehyde (0.4 mol 2-HPP/mol benzaldehyde) in 3 h when applying benzaldehyde and acetaldehyde as substrates in optimized concentrations. Moreover, in this study, it was demonstrated that adding exogenous coenzymes is not needed when BFD is used in *P. putida* resting cells. In total, a cost-efficient strategy to apply whole resting cell catalysts for producing 2-HPP at high concentrations from cheap aldehydes proved to be very effective compared with isolated enzymes.

## Materials and methods

### Chemicals

All the chemicals used in this study were obtained from Merck (Darmstadt, Germany), Sigma (USA), Samchun Chemical Co. (Korea) and were of reagent grade unless otherwise stated.

### Growth and maintenance of bacterial strain

*P. putida* ATCC 12633 (PTCC 1694) was obtained from the Persian Type Culture Collection (PTCC). The strain was activated by transferring to BHI (Brain Heart Infusion) culture broth and incubated for 24 h at 30 °C. It was routinely maintained on Tryptic Soy Agar (TSA) plates and sub-cultured biweekly.

### Whole-cell biotransformation

#### Culture conditions

To induce whole-cell BFD and its carboligase activity, the growth medium of Hegeman [75], known as mandelate medium and containing (in g per liter): ammonium mandelate, 3; nitrilotriacetic acid, 0.2; MgSO_4_.7H_2_O, 0.58; CaCl_2_.2H_2_O, 0.067; (NH4)_6_ MO_7_O_24_.4H_2_O, 0.0002; FeSO_4_.7H_2_O, 0.002; KH_2_PO_4,_ 3.4; Na_2_HPO_4_.12H_2_O, 6.7; yeast extract, 1 was used. The final pH was set to 7.0. Racemic (D/L) mandelic acid was used as substrate to induce mandelate pathway. Moreover, phosphate ions are required to accumulate a large amount of BFD enzyme in cells [61]. Ammonium mandelate is a mixture of 1 mL ammonium hydroxide [ammonia solution 25% (v/v), Merck] and 0.36 g of D/L-mandelate. Carbon and energy sources such as inducer and yeast extract should be autoclaved separately at 121 °C for 15 min. Finally, they were added to other mineral solutions before bacterial inoculation. Bacterial colonies from TSA stock cultures were loop inoculated in a 250 mL Erlenmeyer flask containing 50 mL mandelate medium and incubated at 30 °C on an orbital shaker set at 200 rpm. The cell growth was monitored by measuring turbidity and its optical density reading at a wavelength of 600 nm. To prepare completely induced bacteria for biotransformation, seed cultures were grown overnight until the late exponential phase of growth in a 250 mL Erlenmeyer flask containing 50 mL mandelate medium. The OD_600_ value of this culture was maintained at 1.6 to 1.7 and then transferred into a 2-l flask containing 500 mL fresh medium at an inoculum rate of 10% (v/v) and incubated at 30 °C and 200 rpm for 3–4 h, to the mid-exponential phase of growth when a cell turbidity (OD_600_) of 0.8–0.9 was obtained. Afterward, all the cells were harvested by centrifugation (10,000 × g for 10 min at 4 °C) in 50 mL tubes followed by washing with sodium phosphate buffer (50 mM, pH 6) twice and then re-centrifuging. The supernatant was removed and the pellet was used as the whole resting cell biocatalysts. The pellets were frozen at -20 °C and thawed when needed.

### Resting cell suspension

The biotransformation reaction using benzaldehyde and acetaldehyde as substrates was carried out. Two different experiments were set up. First, a benzoin condensation reaction was performed at a fixed concentration of benzaldehyde and with different acetaldehyde concentrations (between 200 and 1600 mM) by resuspended cells in a volume of 6 mL phosphate buffer inside a 100 mL Erlenmeyer flask. In all cases for running the reactions, washed bacteria with a known wet weight of cells were directly resuspended in sodium phosphate buffer, 200 mM and pH 7, which is the optimum pH for BFD-catalyzed carboligation with benzaldehyde as a donor substrate [15, 59]. These cell suspensions were referred to as resting cells. The reaction was continued for 25 h. Samples were taken every 5 h and immediately analyzed. The optical density of the suspension at 600 nm was adjusted to 20 with plain buffer as blank which is equivalent to 0.033 WCW/mL of buffer or 0.006 DCW/mL. Due to the low solubility of benzaldehyde in aqueous medium and the formation of a benzaldehyde-aqueous two-phase system in concentrations beyond its maximum solubility (50 mM; 5.3 g L^− 1^), benzaldehyde at a fixed concentration of 50 mM was used. Higher concentrations exceed the water solubility of benzaldehyde, which can inactivate the enzyme [76]. All reactions were performed in aqueous monophasic media. No cofactors were added externally throughout the whole process development. 2-HPP formation from the biotransformation reaction was monitored over time by gas chromatography (GC) analysis. Therefore, by determining optimal acetaldehyde concentration, the profile of 2-HPP formation by a fixed amount of whole cells (0.033 g WCW/mL) was studied. The reaction was run at regular intervals in 6 mL reaction volume in a 100 mL Erlenmeyer flask. The effect of *P. putida* cell concentration on the production of 2-HPP was investigated using fixed amounts of benzaldehyde (50 mM) and acetaldehyde (600 mM) over 3 h. Different quantities of wet cells from 0.016 to 0.83 g WCW/mL were added as catalyst. The values of optical density at 600 nm were used to calculate the cell mass. OD_600_ of 10 was approximately equivalent to 16 g L^− 1^ of wet weight and 3 g L^− 1^ of dry cell.

In separate, but comparable biotransformation, the reaction was run at a fixed concentration of acetaldehyde (600 mM; 26.43 g L^− 1^) and different benzaldehyde concentrations (10, 20, 30, 40, 50 mM) in 200 mM phosphate buffer at pH 7, 30 °C and 200 rpm with a cell concentration of 0.033 g WCW/mL. The reaction was continued for 3 h and the concentrations of substrates and products at different time intervals were analyzed by GC.

The effect of pH on the biotransformation was investigated at pH values of 4, 5, 6, 7, 8 and 9.

### Analytical techniques

After the biotransformation, cells were removed by centrifugation at 10,000 ×g for 10 min. The resulting supernatant was vortexed vigorously for 10 min with the same volume of dichloromethane and centrifuged for another 10 min at 20,817 ×g at 4 °C for complete separation of two phases. The concentration of S-2-HPP, benzyl alcohol and remaining benzaldehyde were determined by injecting 1 µl sample into a GC apparatus (Agilent 6890) equipped with an FID detector. The GC contained an HP-5 capillary column (30 m × 0.25 mm i.d., film thickness 0.25 μm). Helium was used as the carrier gas with a flow rate of 1 mL/min. The injector temperature and detector temperature were set at 180 °C and 240 °C, respectively. The oven temperature was initially 80 °C and then increased to 150 °C at a rate of 5 °C/min and remained for 2 min at this temperature. Authenticate samples of 2-HPP, benzaldehyde and benzyl alcohol (a potential by-product from benzaldehyde) were initially used as injection standards, so standard curves were plotted separately for each analysis run. The retention times of benzaldehyde, benzyl alcohol and 2-HPP were 5.14, 6.28., and 11.11 min, respectively. Final confirmation of the chemical structure of benzaldehyde, benzyl alcohol and 2-HPP was done by employing GC-MS.

### Immobilization of ***P. putida*** resting cells in alginate beads

A modified alginate composite was adopted and used for immobilization of cells [77]. Briefly, PVA (2% (w/v)) and sodium alginate (4% (w/v)) were dissolved in distilled water by shaking on a magnetic stirrer for 30 min and sterilized at 121 °C for 15 min. After cooling, CaCO_3_ solution (3%) was added to the mixture at a ratio of 1:3 v/v and mixed thoroughly until a homogenous solution was obtained. Three g WCW of bacterial cells, harvested by centrifuging from mandelate medium, was added to 35 mL of sodium alginate-PVA solution. This mixture was dripped via a 20 mL syringe through a thin needle into 200 mL of cold CaCl_2_·2H_2_O 2% (w/v) and saturated (6% w/v) boric acid. The distance between needle tip and the surface of solution was 6–7 cm to have a better bead size and shape. The droplets formed gel spheres instantaneously and cells were entrapped inside the cross-linked alginates. The beads were kept at 4 °C for 24 h to complete the crosslinking process and gain a uniform spherical shape. The average bead diameter was nearly 2 mm and the encapsulation efficiency was 99%. Finally, the beads were washed with CaCl_2_ 2% (w/v) several times to remove any remaining BA, sodium alginate, and PVA. Sterilized conditions were maintained throughout the procedure.

### 2-HPP production by encapsulated whole cells

Two-hundred capsules were added to 6 mL of 200 mM succinate buffer containing 5 g L^-1^ CaCl_2_  at pH 7 (equivalent to 0.033 g WCW/mL) in a 100 mL Erlenmeyer flask containing benzaldehyde (20 mM and 40 mM) and acetaldehyde (600 mM). The beads remain stable without being dissolved in succinate-NaOH buffer. Biotransformation with immobilized cells was carried out at 30 °C and 200 rpm for 5 h.

### Reuse and reactivation of the immobilized cells

After completion of each biotransformation cycle, beads were collected and washed with CaCl_2_ 2% (w/v) and sterile deionized water and used again for the next cycle. The concentration of 2-HPP production was determined by GC. Appropriate controls with similar conditions but with free cells were run simultaneously.

## Data Availability

The datasets generated during and/or analyzed during the current study are included in this published article. They are also available from the corresponding author on a reasonable request.
